# Biosynthesis and Anti-inflammatory Activity of Zinc Oxide Nanoparticles Using Leaf Extract of *Senecio chrysanthemoides*

**DOI:** 10.1155/2023/3280708

**Published:** 2023-04-11

**Authors:** Sana Zahoor, Sadia Sheraz, Dilawar Farhan Shams, Gauhar Rehman, Saira Nayab, Muhammad Ishaq Ali Shah, Muhammad Ateeq, Said Karim Shah, Tanveer Ahmad, Sulaiman Shams, Waliullah Khan

**Affiliations:** ^1^Department of Chemistry, Abdul Wali Khan University, Mardan, Pakistan; ^2^Department of Environmental Sciences, Abdul Wali Khan University, Mardan, Pakistan; ^3^Department of Zoology, Abdul Wali Khan University, Mardan, Pakistan; ^4^Department of Chemistry, Shaheed Benazir Bhutto University, Sheringal Dir (U), Pakistan; ^5^Department of Physics, Abdul Wali Khan University, Mardan, Pakistan; ^6^Department of Biochemistry, Abdul Wali Khan University, Mardan, Pakistan

## Abstract

Nanotechnology has recently appeared as an important study subject in modern material sciences. Greener synthesis of nanoparticles has gained the attention of many scientists because of its integral characteristics such as effectiveness, eco-friendly, and low cost. In the present study by following the green synthesis approach, zinc oxide nanoparticles (ZnO NPs) were formed for the very first time by using *Senecio chrysanthemoides* leaf extract as a reducing agent. The UV-Vis spectrophotometer was used to study the synthesized ZnO NPs, and the specific peak was found to be at 349 nm. The characteristic Fourier transform infrared (FTIR) peak was found to be at 449 cm^−1^ which displays the peak of ZnO molecules. The surface morphology of the ZnO NPs was determined via scanning electron microscopy (SEM). The energy-dispersive X-ray spectroscopy (EDX) study showed that the synthesized ZnO NPs are present at the weight percentage of 66.38%. The X-ray diffraction (XRD) spectrum confirmed the hexagonal phase wurtzite structure, with the average particle size of 31 nm, and demonstrated the crystalline structure of ZnO NPs. Additionally, to all these experiments, we compared the anti-inflammatory properties of biogenic ZnO NPs to a standard drug. Biosynthesized ZnO NPs have revealed an effective anti-inflammatory activity at a higher concentration (100 mL^−1^) and showed 73% inhibition in comparison with diclofenac sodium drug. Zinc oxide was shown to be compatible with diclofenac sodium, according to the results. The ZnO NPs produced using the greener synthesis process have the potential to be used in a broad range of fields and also used as a good anti-inflammatory agent.

## 1. Introduction

Nowadays, nanotechnology is one of the booming fields for researchers. There is a wide variety of uses for nanomaterials because of their small size and shape making them an important topic in both basic and applied sciences. Nanomaterials with relevant features such as size ranging from 10 to 100 nm can be produced using a variety of approaches. Nanosized semiconductors have become a significant topic in recent times because of their unique features that can be used in optoelectronics [1]. Microscopic particles smaller than 100 nm are known as nanoparticles. Because they bridge the gap between bulk counterparts as well as atomic or molecular structures, nanoparticle study is a dynamic topic in science and technology.

When examined at the nanoscale, a number of well-known bulk materials have revealed surprising features. The large aspect ratio of nanoparticles is one of many explanations for this [2]. In the field of biomedicine, nanoparticles of metal oxides (MONPs) have been introduced, specifically for antibacterial, anticancer, gene transport, biosensing, and cell imaging applications [3]. In recent decades, the significance of these nanoparticles has increased due to their exceptional chemical and thermal stability. Zinc (Zn) and its oxide constitute one of the most intriguing and potentially rewarding metallic nanomaterials (ZnO). Zinc is one of the microelements that are absolutely necessary for the survival of living organisms [4]. Zinc is a highly active element while at the same time a powerful reducing agent; in accordance with its reduction potential, it can effortlessly oxidize, leading to the formation of zinc oxide, which is also very useful in the preparation of ZnO NPs [5]. Zinc oxide nanoparticles, sometimes referred to as ZnO NPs, are semiconductors that stand out from the crowd when compared to other nanoparticles due to their distinctive characteristics. The development of the optoelectronics sector is based on these features in several ways [6]. The preparation of ZnO NPs by varying shape and size, in accordance with the purpose for which they are required, involves the employment of a number of different chemical and physical approaches. Sol-gel, thermal, microwave-assisted chemical vapor deposition, electrochemical reactions, infrared irradiation, pulsed laser deposition, and sputtering are the methods that are being utilized frequently [7]. Chemical and physical approaches are comparatively expensive and require critical conditions of temperature, pressure, time, and energy consumers, as well as chemicals required in the procedure harmfully disturb the environment, so these methods have their own set of drawbacks to the environment. A technique known as “green synthesis” uses microbes, for example, bacteria and yeast, along plant extracts to synthesize cost-effective and effective nanoparticles [8, 9]. Researchers are concerned about the environment, helping them to focus their attention towards eco-friendly synthesis methods [10]; due to this concern, it has been possible now to produce ZnO NPs by employing a precursor of Zn(II) in the presence of plant extracts [11] as cost-effective environmental friendly approach. Biotechnological ZnO nanoparticles have been synthesized using a variety of plants and their extracts, including *ginseng* and *Rhodiola rosea* [12]. When it comes to the synthesis of nanoparticles, biological systems play a critical and flexible role as capping agents, stabilizing the nanoparticles and allowing the production of specific-sized and specific-shaped nanoparticles. Using plant fragments such as roots, leaves, and stems has several benefits, plants are readily accessible and safe to handle, and the nanoparticles made using plant extracts are also more stable [13, 14].

ZnO nanoparticles are identified as multifunctional, basic, and nontoxic inorganic material. It is inexpensive and has a broad range of applications in various fields due to its versatile nature, for example, delivery of drugs and antimicrobial agent as well as bio-imaging, anti-inflammatory agent, and wound healing [15, 16]. The additional benefit of the green synthesized NPs is that they could also be used in biological applications such as anti-inflammatory activities of metal oxides without any harmful impacts [17]. Even though ZnO NPs can be useful in a variety of fields, they can also be toxic to organisms. Cobalt, copper, iron, nickel, and zinc all have the potential to cause different diseases. It is possible that ZnO NPs released into aquatic ecosystems through sewage treatment plants and other sources could have harmful impacts on fish and other aquatic creatures [18, 19]. A disease known as zinc fever can occur when zinc powder is inhaled or eaten, resulting in symptoms such as chills, fever, and coughing. In order to serve humanity, various goods that contain ZnO nanoparticles are being developed, and the demand to produce them is expanding every day. These nanoparticles were formed utilizing the *Senecio chrysanthemoides* leaf extract in this study. Green synthesis approaches help in ZnO nanoparticles manufacturing in a way that is favorable to the environment, produces less waste, and is extremely safe and nontoxic. A variety of characterization approaches have been used to determine the properties and structure of the NPs produced. The anti-inflammatory analysis of nanoparticles was studied using a heat-induced hemolysis process against standard drugs.

## 2. Results

### 2.1. Characterization of Synthesized ZnO NPs

**Figure 1:**
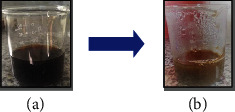
Preliminary observation of prepared ZnO NPs: (a) initial color and (b) final color change.

**Figure 2:**
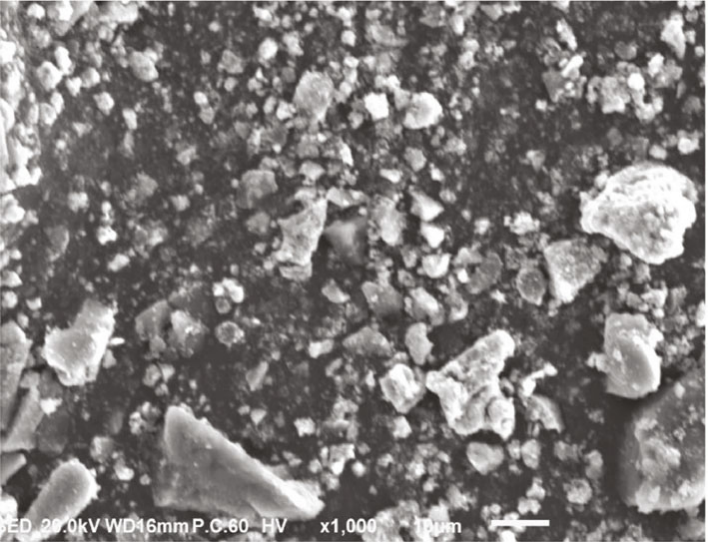
SEM images of ZnO NPs.

**Figure 3:**
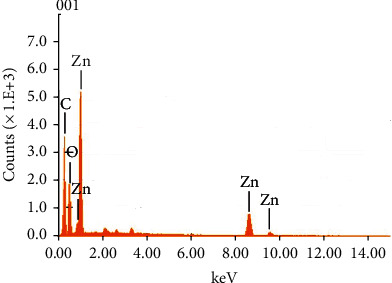
EDX spectrum of ZnO NPs.

ZnO NPs have drawn a lot of attention owing to their outstanding optical properties. The visible color shift is an early indicator of metal reduction and nanoparticle production.  Figure 1 shows the synthesis of ZnO NPs using freshly obtained *Senecio chrysanthemoides* leaf extracts [20]. Biogenically produced ZnO NPs were characterized via SEM, FT-IR, UV-Vis, XRD, and EDX techniques. The surface morphology was examined with the use of scanning electron microscopy. The SEM image of the ZnO NPs as prepared is displayed in Figure 2 which shows crushed-ice shape morphology. In a study, it was found that the surface morphology of prepared ZnO powders was measured using the SEM analytical technique, showing a crushed-ice shape with homogenous size distributions for ZnO NPs [21]. The EDX spectrum was used to analyze the elemental composition of ZnO NPs produced from *Senecio chrysanthemoides* leaf extract (Figure 3). The formed ZnO NPs existed in a weight percentage of 66.38% and 33.62%, according to the EDX analysis.

**Figure 4:**
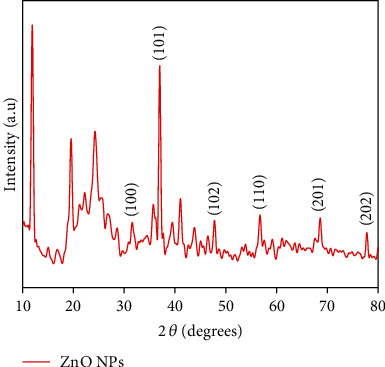
XRD pattern of ZnO NPs.

The green synthesized ZnO NP pattern of XRD was observed between 20° and 80° 2*Ө* range. Pattern of XRD of ZnO NPs was matched to the standard dataset provided by the JCPDS, as shown in Figure 4. The XRD spectra of ZnO NPs showed a strong diffraction peak corresponding to the 2*Ө* peaks that are characteristic of ZnO NPs that may be seen at 31, 35, 37, 47, 56, 68, and 77, and they correspond to the (100), (002), (101), (102), (110), (201), and (202) planes of the crystal lattice; correspondingly, structure of wurtzite being hexagonal in shape and broadening of the peaks at the bottom are indications that the crystalline sizes are small.

**Figure 5:**
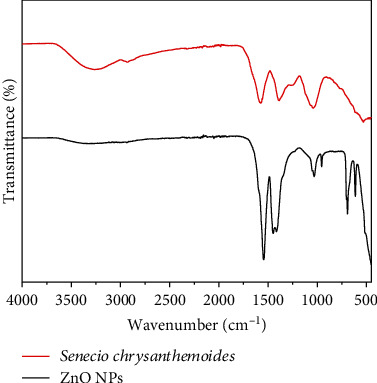
FTIR spectra of *Senecio chrysanthemoides* plant and ZnO NPs.

**Figure 6:**
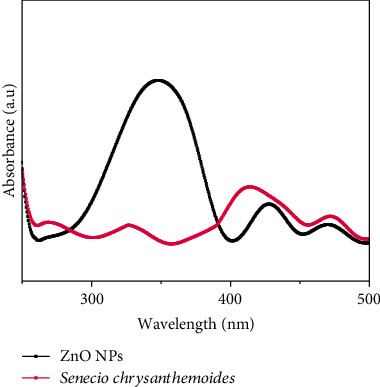
UV-Vis spectra of ZnO and *Senecio chrysanthemoides* plant.

In order to determine which functional groups exist in the *Senecio chrysanthemoides* leaf extract that contributed to the stabilizing process for ZnO NPs, FT-IR analysis was performed on the sample.  Figure 5 shows the FTIR absorption spectra between 4000 and 400 cm^−1^. Broad absorption peaks are found at 3269, 2943, 1544, 1393, 1051, and 530 for *Senecio chrysanthemoides* leaf extract. The FTIR spectra of ZnO NPs displayed numerous absorption peaks at 3269, 2959, 1544, 1439, 1032, 955, 692, 612, and 449 cm^−1^ [22].  Figure 6 illustrates the UV-Vis spectra of *Senecio chrysanthemoides* leaf extract and ZnO NPs. An ultraviolet-visible spectroscopic study was performed in order to ensure the biogenesis of ZnO NPs. In order to carry out this typical analysis, the sample was initially diluted in deionized water. The range of wavelengths from UV-visible was between 200 and 800 nm. The strong peak obtained at 349 nm revealed the presence of ZnO NPs in the mixture. It is possible that the movement of the electronic cloud on the total skeleton of the ZnO NPs is responsible for the broad absorption band that extends towards longer wavelengths. UV-Vis analysis was also conducted for the plant extract plus the results indicated many peaks at various wavelengths in the range of 200-550 nm [23]. The stability of ZnO NPs was studied against heat, salt concentration, and pH. The ZnO NPs were found to be highly stable at 80°C, 10 mMol concentrations of NaCl, and in 3-11 pH range.

### 2.2. Anti-inflammatory Activity of Leaf Extract and Zinc Oxide Nanoparticles

#### 2.2.1. Heat-Induced Hemolysis Assay

Inflammation is an automatic response that is displayed by the immune system of the body in response to a wide variety of bacteria, irritants, damaged cells, and harmful stimuli. Numerous secondary metabolites plus a variety of metallic nanoparticles have been shown to possess anti-inflammatory properties; these properties have been demonstrated both in vitro and in vivo [24]. The effectiveness of ZnO NPs as an anti-inflammatory agent was evaluated in vitro through the use of heat-induced hemolysis. This test's methodology is predicated on the stability as well as lysis of RBC membranes as the principle of investigation. With the help of RBC membrane lysis inhibition, the HRBC strength of MGE was evaluated while the experiment was carried out at high temperature.

**Figure 7:**
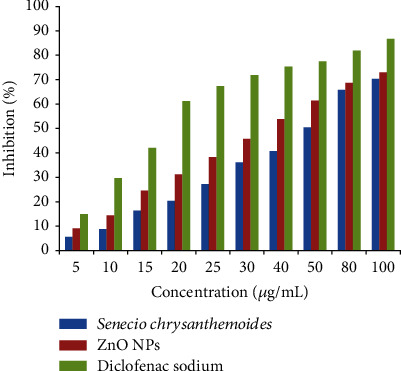
View of the images of the anti-inflammatory analysis of *Senecio chrysanthemoides* leaf extract and ZnO NPs against standard drugs.

Zinc oxide NPs and *Senecio chrysanthemoides* leaf extract were used at concentrations such as 5, 10, 15, 20, 25, 30, 40, 50, 60, 70, 80, and 100 *μ*g/mL (Figure 7), with absorbance values of 9.05, 14.47, 24.57, 31.23, 38.28, 45.71, 53.9, 61.38, 65.71, and 68.31 percent and absorbance value of leaf extract of 5.65, 8.82, 16.36, 20.37, 27.27, 36.17, 40.72, 50.52, 65.82, and 70.31, correspondingly. We used 10 mg/10 mL of diclofenac sodium drug as a reference as 5, 10, 15, 20, 25, 30, 40, 50, 60, 70, 80, plus 100 *μ*g/mL, which exhibited 14.9, 29.66, 42.04, 61.23, 67.42, 71.8, 75.38, 77.38, 81.95, plus 86.71% absorption. The least absorption rate of ZnO NPs was 9.05 percent and that of plant leaf extract was 5.65 percent at 5 *μ*g/mL, while the maximum absorption rate of ZnO NPs was 73.01 percent and that of leaf extract was 70.31% at 100 *μ*g/mL (Table 1).

## 3. Materials and Methods

### 3.1. Chemicals

All the chemicals, reagents, and solvents used in this study were of analytical grade from Sigma-Aldrich (Germany). The plant sample was found and collected from Kumrat Valley, Dir Upper, Khyber Pakhtunkhwa, Pakistan. Standard drug from the Zoology Lab of Abdul Wali Khan University Mardan, Pakistan, was taken.

### 3.2. Preparation of *Senecio chrysanthemoides* Leaf Extract

The leaves of *Senecio chrysanthemoides* plant were cleaned twice with distilled water to eradicate dirt before drying in the shade for 3-4 days. A grinder was used to grind the dried leaves into a fine powder, which was stored in zip mouth polyethylene bags in the shade for future use. 6.34 g of powdered leaves was added to a 150 mL beaker of distilled water then heated with 100 mL of distilled water for about 40 min at 80°C, then allowed to cool at ambient temperature (approximately 25°C). The Whatman No. 1 filter paper was used two times to filter the solution completely.

### 3.3. Synthesis of Zinc Oxide Nanoparticles

To make ZnO NPs, zinc acetate-2-hydrate with chemical formula Zn(CH_3_COO)_2_·2H_2_O has been used. 20 mL of *Senecio chrysanthemoides* plant's leaves was extracted, and then, 1 g of zinc acetate-2-hydrate salt was added. The initial color of the solution was dark brown. A stirrer plate assembly was used to constantly stir the solution at 70°C for approximately 60 minutes. When zinc ions are reduced, a thick precipitate was formed in the solution. This precipitate was centrifuged at 6000 rpm for 25 minutes, the upper phase was decanted, and the residual part was washed twice with distilled water and methanol, and thus, the resulting solid part (ZnO NPs) was desiccated in the oven at 110°C for 24 hours. The NPs were calcined in a furnace at 400°C for two hours to improve their crystallinity. ZnO NPs were discovered by utilizing a UV-Vis spectrophotometer to look for particle size ranging from 200 to 800 nm. Afterwards, the NPs were deposited in glass vials for more characterization along with other operations.

### 3.4. Characterization of ZnO NPs

With the UV-Shimadzu-800 (Japan) equipment, the synthesis of ZnO NPs was characterized via measuring the UV-Vis absorption spectrum between 200 and 800 nm. When *Senecio chrysanthemoides* leaf extract-mediated ZnO NPs were studied on a spectrometer called Perkin Elmer Spectrum in the 400–4000 cm^−1^ range, FT-IR analysis was used to identify the presence of phytochemicals. As part of this study, an X-ray generator equipped named the JEOL Japan JDX-3532 X-ray by Copper K*α* radiation (40 kV and 1.5406 A°) was used to generate measurements of the crystal structure of nanoparticles using XRD. Using a Hitachi SEM (Hitachi S 4800, Tokyo, Japan), the morphology of ZnO NPs was studied under 10 kV. An electron beam strikes the sample's surface with the EDAX process to examine the elemental composition of ZnO NPs (SEM). 0 to 14 keV is the range of the beam's energy. Exposure to radiation causes it to release X-rays. The X-rays' energy is determined by the materials being studied in the X-ray tube. X-rays are generated at a depth of 2 *μ*. After passing the electron beam through the samples, a 2-dimension picture of each component can be retrieved.

### 3.5. Biological Activities

#### 3.5.1. Anti-inflammatory Analysis of Leaf Extract and Zinc Oxide Nanoparticles

Human red blood cells (HRBCs) were used to study the anti-inflammatory impact of ZnO NPs using the heat-induced hemolysis method [24]. Diclofenac sodium (10 mg/10 mL) was used in PBS for this activity (pH 7.4). A healthy participant provided a blood sample of approximately 5 mL that was NSAIDS-free. Blood was injected into the flacon tube and then centrifuged at 3000 rpm for approximately 20 minutes adding EDTA as an anticoagulant. To remove the supernatant, the remaining components were segregated. Iso-saline solutions (*w*/*v*) were used to clean the remnants. Centrifugation and washing are repeated thrice until a clear supernatant has been achieved. Isotonic saline solution was utilized to generate a 10% suspension of HRBC pellets in solution. To prepare the control, standard, and test samples, the following reactions were carried out. Control reaction mixtures included 100 microliters of blood suspension of 10% and 900 microliters (distilled water of 20 microliters and PBS solution of 800 microliters). Standard reaction mixtures were used to make 100 microliters of blood suspension of 10% and different ratios of diclofenac sodium (DS) in PBS solution (20 microliters of DS plus 880 microliters of PBS, 40 microliters of DS plus 860 microliters of PBS, 60 microliters of DS plus 840 microliters of PBS, and 80 microliters of DS plus 820 microliters of PBS). Various ratios of EGE utilizing PBS solution (20 microliters of MGE plus 880 microliters of PBS, 40 microliters of MGE plus 860 microliters of PBS, 60 microliters of MGE plus 840 microliters of PBS, and 80 microliters of MGE plus 820 microliters of PBS) are contained by reaction mixture in 100 microliters of 10% blood suspension. Incubation at 54°C for 30 min was followed via centrifugation at 5400 rpm for approximately 10 minutes for each test. All samples were read in thrice. Spectrophotometers were used to measure the absorbance of all samples.

The formula for calculating the percentage of inhibition of HRBC lysis is as follows:

(1) $$\begin{array}{c} \%\text{Inhibition}=\frac{\text{Control Abs}-\text{Sample Abs }}{\text{Absorbance of control}}\times 100. \end{array}$$

## 4. Discussion

As an alternative to chemical and physical approaches, the use of plant extracts in the synthesis of NPs has recently attracted a lot of attention. Chemicals that are costly and harsh are eliminated through the use of bioactive compounds from plants. Extracellular or intracellular biological components from plants can be used to synthesize NPs. In the current study, the aqueous Zn^2+^ was reduced to ZnO NPs when added to leaf extract derived from the *Senecio chrysanthemoides* plant. It was proposed that biological substances secreted into the reaction mixture by plant extract and their functional group caused the reduction of Zn^2+^ to ZnO NPs. Following the addition of plant extract to zinc acetate dihydrate and heating at 70°C, preliminary confirms ZnO NP production. One initial indicator of ZnO NP biological production could be a change in color of the reaction mixture. The selected plant extracts' phenolic and flavonoid content is assumed to be the cause of the ZnO reduction. The UV-Vis spectroscopy at 349 nm provided strong evidence that ZnO NPs were synthesized. Similarly, earlier research on the production of ZnO NPs using plant extracts (e.g., *pomegranate fruit peel* and *solid coffee grounds*) reported the same range of absorption peaks [25].

The structure, size, and elemental composition of the synthesized ZnO NPs were determined using the SEM, XRD, and EDX. SEM images exhibited that the produced biogenic ZnO NPs have a crushed-ice shape morphology indicating that the used extracts had good capping and stability capabilities. The pristine and crystallinity of the prepared nanoparticles are shown through the strong and narrow peak diffraction in the XRD spectrum. The size of crystallinity of the synthesized NPs can be calculated by applying Debye-Scherrer's formula. The formula for Scherrer is given as follows:

(2) $$\begin{array}{c} D=\frac{K\lambda }{\beta \cos\theta }, \end{array}$$

where *D* denotes the crystallite size, *λ* denotes the wavelength of the X-ray that was employed (1.5406 Ǻ), *β* denotes the whole width measured at its maximum width in half (FWHM), and theta denotes Bragg's angle. The prepared ZnO nanoparticles, on average, are 31 nm in size. By literature study, sharp diffraction peak with 2 theta values was found to be at 31.80°, 34.45°, 36.28°, 47.59°, 56.65°, 62.94°, 66.46°, 68.00°, 69.09°, 72.57°, and 77.0648° according to the results of XRD characterization measurements in a report, in which ZnO NPs were synthesized from aqueous extracts of Deverra tortuosa through greener approach. The peaks at 100, 002, 101, 102, 110, 103, 200, 112, 201, 004, and 202 confirm that they are hexagonal wurtzite lattice planes [26]. In another study, similar results were studied [27]. Based on EDX analysis, a strong peak of zinc atoms as well as a peak produced by O may be seen, whereas C atoms indicate the contribution of biomolecules present in the extract. The researchers discovered three peaks of zinc emission at 38.88 percent and one emission peak of C and O at 42.50 percent and 15.90 percent, respectively; in another study, in which ZnO NPs were synthesized via cell extract, indication of the presence of these compounds in the cell extract formed ZnO NPs [28].

The leaf extract of *Senecio chrysanthemoides* contains a high concentration of polyphenolic derivatives, which are believed to be biologically active chemicals that might be used in medical therapy and also act as reducing agents. The FTIR spectrum of the extract of plant is contrasted to that of the green synthesized ZnO nanoparticles in Figure 6. As a result of this, the FTIR spectra of the extract contained numerous peaks at 3269 (O-H), 2943 (C-H), 1544 (C=C), 1393 (C-N), 1051 (C-O), and 530 (C-I) cm^−1^. These peaks belong to alkaloids, flavonoids, and phenolic chemicals, correspondingly. According to these observations, the extract of *Senecio chrysanthemoides* leaf contains flavonoid derivatives. Functional group interaction of flavonoids as well as phenols along with the ZnO NPs may be responsible for the shifts or changes in the position of the peaks in the sample spectrum. It is important to note that the electrons, given by the functional groups of extract, can result in a drop in zinc ions (Zn^+2^ ⟶ Zn^1+^) and also in zinc NPs (ZnO). In addition, the negative functional groups that are present in the extract might have a stabilizing property. Indication of the primary biomolecules from the extract was also capped or connected to the ZnO NP surface; the FT-IR spectrum of the biosynthesized ZnO NPs indicated a slight shift along with minor alterations in various related peaks as well as intensities. Stretching of vibrations for the O–H groups in water, alcohol, and phenols is responsible for the absorption bands in ZnO NPs' FTIR spectra at 3269 cm^−1^. C=C stretch in aromatic rings and C=O stretch polyphenols are responsible for the sharp bands at 1544 cm^−1^. The protein present in extract gives the C–N stretch of amide-I a 1439 cm^−1^ band. An amino acid C–O stretching causes a 1032 cm^−1^ band, while C–H bending vibrations peak at 612 and 692 cm^−1^ and C–O-C stretch vibrations peak at 955 cm^−1^. The characteristic peaks of ZnO molecules may be identified in the IR spectrum of the ZnO nanoparticles as additional peaks at 449 cm^−1^. The IR spectra of the prepared ZnO NPs make it abundantly evident that these biomolecules are actively participating in the process of reducing and stabilizing the ZnO NPs. On the other hand, it was observed that the amino acids plus amide linkages in proteins were significant for the stability of the ZnO nanoparticles [29–32]. ZnO nanoparticles are multifunctional elements that are commonly used in many applications. However, its biocompatibility with biological drug is of considerable concern. In light of the results obtained from anti-inflammatory activity, green synthesized zinc oxide nanoparticles are good anti-inflammatory agents. They can be very important sources in the therapeutic and pharmacological fields to alleviate various diseases.

## 5. Conclusions

This research work presented the biosynthesized ZnO NPs using leaf extract of *Senecio chrysanthemoides*, a well-known plant for its medicinal value. This method is green, harmless, as and simple compared to others. The NPs were synthesized by reducing plant biomolecular agents. In case of ZnO NPs, the color change indicated the synthesis process. XRD analysis has verified that the produced NPs have a crystalline structure. By using FTIR, it was determined whether phytochemicals were present when metallic ions were transformed into nanoparticles. Morphology was established by SEM spectra. The UV-Vis study determined the presence of ZnO NPs in *Senecio chrysanthemoides* leaf extract, with a peak at 349 nm. The presence of zinc and oxide ions in nanoparticles was confirmed by EDX study. The produced ZnO NPs have shown good anti-inflammatory abilities. ZnO NPs have also revealed a moderate inhibitory ability against diclofenac sodium drug. The synthesized ZnO NPs showed more effective anti-inflammatory activity than the *Senecio chrysanthemoides* leaf extract; according to the result, the maximum absorption rate of zinc oxide NPs was 73.01 percent and that of leaf extract was 70.31% at 100 *μ*g/mL. Our results concluded that the above-mentioned ZnO NPs could be considered for application in medicines and pharmaceuticals, owing to their good anti-inflammatory effect and in treatment of various diseases including other inflammatory diseases. Zinc oxide nanoparticles, with which the production and consumption ability are growing, might be provided to humanity in the environment as well as biomedical areas to offer numerous benefits. More study on ZnO NPs is needed to investigate their biological potentials both in vitro and in vivo.

## Figures and Tables

**Table 1:** ZnO NPs and various values of drug.

Serial number	Concentration (*μ*g/mL)	% inhibition		
		Leaf extract	ZnO NPs	Standard
1	5	5.65	9.05	14.9
2	10	8.82	14.47	29.66
3	15	16.36	24.57	42.04
4	20	20.37	31.23	61.23
5	25	27.27	38.28	67.42
6	30	36.17	45.71	71.8
7	40	40.72	53.9	75.38
8	50	50.52	61.38	77.57
9	80	65.82	68.71	81.95
10	100	70.31	73.01	86.71

## Data Availability

All data are included in the manuscript.
